# Antioxidant and antifungal activities of essential oil of *Alpinia calcarata* Roscoe rhizomes

**DOI:** 10.4103/0975-9476.72621

**Published:** 2010

**Authors:** Lakshmi S.R Arambewela, L.D.A. Menuka Arawwawala, Nandakumara Athauda

**Affiliations:** *Industrial Technology Institute, Colombo, Sri Lanka*

**Keywords:** *Alpinia calcarata*, antifungal activity, antioxidant power, essential oil

## Abstract

Antioxidant and antifungal activity were determined for the essential oil of *Alpinia calcarata* Roscoe (Zingiberaceae) rhizomes. Its antioxidant properties were investigated by the 2,2-diphenyl-1-picrylhydrazyl (DPPH) free radical scavenging assay and thiobarbituric acid reactive substances (TBARS) assay. Butylated hydroxy toluene (BHT) and vitamin E served as positive controls. Antifungal activities were investigated against crop pathogens *Curvularia* spp. and *Colletorichum* spp. using the agar plate method. Fifty percent effective concentration (EC_50_) and % antioxidant index of the essential oil were 45 ± 0.4 and 16.1 ± 0.2 for DPPH and TBARS assays, respectively. The degree of, the essential oil’s inhibition of the growth of crop pathogens *Curvularia* spp. and *Colletorichum* spp. varied with time period its effects were higher than greater than for the positive control, daconil. In conclusion, the essential oil of *A. calcarata* rhizomes possess moderate antioxidant property and promising antifungal activity.

## INTRODUCTION

*Alpinia calcarata* Roscoe (Zingiberaceae) is a rhizomatous perennial herb which is commonly used in traditional medicinal systems in Sri Lanka. *A. calcarata* is cultivated in tropical countries including Sri Lanka, India, and Malaysia.[1,2] In Sri Lankan, rhizomes of *A. calcarata*, are recommended as an aphrodisiac, and the decoction is widely used in the treatment of bronchitis, cough, respiratory ailments, diabetics, asthma, and arthritis.[2–4] Some 18 volatile constituents have been identified in the essential oil (EO) of Sri Lankan grown *A. calcarata* rhizomes, of which 1,8-cineol (33.3%) is the major constituent. Apart from 1,8-cineol, α-pinene (3.1%), camphene (4.1%), β-pinene (9.3%), *p*-cymene (1.4%), and limonene (4.0%) are also present.[5]

Research has shown antibacterial,[6] anthelmintic,[7] antifungal,[8] antinociceptive,[9] antioxidant,[10] gastroprotective,[11,12] aphrodisiac,[13] and antidiabetic[14] activities in aqueous and ethanolic extracts of *A. calcarata* rhizomes. However, no attempt has been made to investigate the bioactivities of their EO. Therefore, this study was carried out to investigate its antioxidant power using the 2,2-diphenyl-1-picrylhydrazyl (DPPH) free radical scavenging assay, and the thiobarbituric acid reactive substances (TBARS) assay, and antifungal activity against *Curvularia* spp. and *Colletorichum* spp.

## MATERIALS AND METHODS

### Plant material

Fresh *A. calcarata* rhizomes were collected from home gardens in Western province, Sri Lanka. The plant material was identified and authenticated by the Curator of the National Herbarium, Royal Botanical Gardens, Peradeniya, Sri Lanka. A voucher specimen (AS 01) was deposited in the Industrial Technology Institute, Colombo 7, Sri Lanka.

### Preparation of the essential oil

Fresh *A. calcarata* rhizomes were sterilized with 1% NaOCl, rinsed with sterile distilled water and hydrodistilled for 4 h using a Clevenger arm. The EO was trapped in a mixture of *n*-pentane and ether (1:1 v/v), collected and weighed after evaporating the organic layer (yield 1.5 % w/w dry weight basis).

### Standardization of the essential oil

Gas chromatographic profile was used to standardize the EO.

### Details of the gas chromatograph operating conditions

Chromatograph: HewlettPackard 5890 Series II

Detector: Flame ionization detector

Column: DB-5 MS capillary column (30 m × 0.25 mm id, 0.25 μm film)

Initial oven temperature: 40° C

Final oven temperature: 280° C

Program rate: 10° C/min

Peaks were identified using retention time data, peak enhancement method using authentic compounds, and by comparing their mass spectra with spectra in the data bank. NMR data were obtained wherever possible.

### Determination of antioxidant property of essential oil by DPPH scavenging assay

The antioxidant property was determined by measuring the remaining concentration of DPPH as described by Singh and coworkers.[15] For this assay, known concentrations of (0–100 μg/mL) EO and butylated hydroxy toluene (BHT) were placed in different test tubes. The volume was adjusted to 1 mL by adding methanol (MeOH). Five milliliters of methanolic solution of DPPH (2 mg/100 mL MeOH) were added to these tubes and shaken vigorously. The tubes were allowed to stand at room temperature for 20 min and the absorbance was measured at λ 517 nm (UV-160, Shimadzu, Japan). A control was prepared as above by adding MeOH instead of test solutions. BHT served as the positive control. This experiment was done twice, each time in triplicate.

The DPPH concentration in the reaction medium was calculated from a calibration curve analyzed by linear regression. The percentage of remaining DPPH (%DPPH_REM_) of each concentration was calculated as follows:

$$\%\;{\mathrm{DPPH}}_{\mathrm{REM}}\;=\;{\left[\mathrm{DPPH}\right]}_{T}/{\left[\mathrm{DPPH}\right]}_{C}\times 100$$

where T is the experimental duration time 20 min and C is the control. The mean effective scavenging concentrations (EC_50_) were calculated by plotting the %DPPH _REM_concentrations versus the concentrations of extract used.

### Determination of antioxidant property of essential oil by TBARS assay

The antioxidant property was determined by measuring the oxidation of egg yolk lipids as described by Dorman and coworkers.[16] In this assay, the egg yolk (10%, v/v) solution was prepared in KCl (1.15%, w/v). It was homogenized for 30 sec and ultrasonicated for 5 min and stored at 4 °C until use. The EO, vitamin E, and BHT at 0.01% (w/v) concentrations were prepared using 8.1 % (w/v) sodium dodecyl sulphate (SDS) solution. A solution of 0.8% (w/v) thiobarbituric acid (TBA) was prepared in 1.1% (w/v) of SDS solution. Test solutions (0.1 mL) were added to tubes containing 0.5 mL egg yolk homogenate. After adding 1.5 mL of acetic acid (20%, v/v), the pH value was adjusted to 3.5 with 1 Mol NaOH. Then, 1.5 mL of 0.8% TBA was added and the final volume adjusted to 4 mL with deionized water. Samples were vortexed and left in a 95°C water bath for 60 min. When they had cooled, 5 mL of *n* -butanol was added, vortexed, centrifuged, and the absorbance of butanol layer was measured at λ 532 nm (UV-160, Shimadsu, Japan) against an *n*-butanol blank. The above procedure was followed for the control by using 0.1 mL of 8.1 % (w/v) SDS instead of the test solution. Both vitamin E and BHT served as positive controls. The experiment was done twice, each time in triplicate.

Antioxidant index percentage (AI %) was calculated using the following formula:

$$\mathrm{AI}\;\%\;=\;\left(\mathrm{(1}\;-\;T/\mathrm{C)}\right)\;\times \;100$$

where, *T* = the absorbance of the test sample

*C* = the absorbance of the fully oxidized control

### Determination of antifungal activity of essential oil by agar plate method

In this assay, 1000, 1500, 2000, and 2500 ppm of *A. calcarata* EO were tested against the activity of crop pathogens *Curvularia* spp. and *Colletorichum* spp. Daconil was used as the positive control. The authentication of *Curvularia* spp. and *Colletorichum* spp. were done by polymerase chain reaction (PCR) technique using molecular detection of these fungi. Stock solution of EO was prepared with 95% ethanol. Test solutions were added to potato dextrose agar (PDA) medium, with the temperature of the sterilized medium at 45° C. Circular fungi culture discs were kept in the center of the each plate and the diameter of the fungal culture was measured after 3, 4, 5, 6 and 7 days. Untreated PDA medium served as the control and each experiment was done in triplicate. Growth inhibition percentage was calculated by the equation (*C – T/C*) × 100 where *C* is hyphal extension (mm) of controls and *T* is hyphal extension (mm) of EO-treated plates.

### Statistical analysis

Statistical comparisons were made using a one-way ANOVA test.

## RESULTS AND DISCUSSION

**Figure 1:**
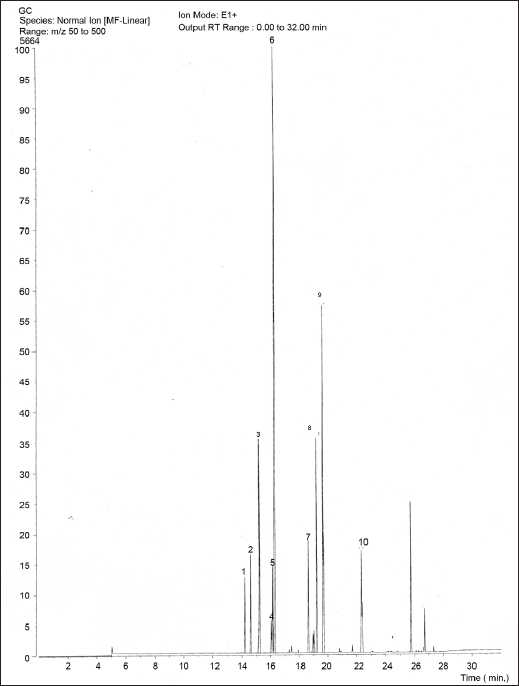
Gas chromatograph of essential oil (EO) of *Alpinia calcarata* rhizomes P eak no 1: α-pinene, peak no 6: 1,8-cineol, peak no 2: camphene, peak no 7: camphor, peak no 3: β-pinene, peak no 8: γ-muurolene, peak no 4: ρ-cymene, peak no 9: caratol, peak no 5: limonene, peak no 10: α-eudesmol

As shown in Figure 1, the EO of *A. calcarata* rhizomes was standardized using a gas chromatograph. Similar to the previous studies,[5] 1,8-cineol was found to be the major component in the EO of *A. calcarata* rhizomes. Antioxidant property of cold ethanolic extract (CEE), hot ethanolic extract (HEE), and hot water extract (HWE) of *A. calcarata* rhizomes have been investigated in previous studies. Further, antioxidant property of CEE was comparable to BHT, the synthetic antioxidant.[10] However, in the present investigation, EO of *A. calcarata* rhizomes showed a moderate antioxidant property (as judged by DPPH scavenging assay [Table 1] and TBARS assay [Table 2]) when compared to the antioxidant properties of CEE, HEE, and HWE. Antioxidants react with the free radical DPPH and convert it to 1,1-diphenyl-2-picrylhydrazine by donating a hydrogen.[17] The change in absorbance produced by this reaction was used to test the ability of EO to act as a free radical scavenger. Therefore, this indicates that DPPH radical scavenging activity of EO is due to its hydrogen-donating ability. Lipid peroxidation, which is widely recognized as a primary toxicological event, is caused by the generation of free radicals from a variety of sources including organic hydroperoxides, redox cycling compounds, and iron-containing compounds. The TBARS assay has been used to measure the degree of lipid peroxidation. TBA reacts specifically with malondialdehyde (MDA), a secondary product of lipid peroxidation. to give a red chromogen, which may then be determined spectrophotometrically.[18] The degree of the EO’s inhibition of the growth of the crop pathogens *Curvularia* spp. and *Colletorichum* spp. varied with time period [Table 3]. Its antifungal activity appears to be more pronounced against *Curvularia* spp. and the effect was better than that of positive control, daconil. Therefore, *A. calcarata* essential oil can be used to prepare a natural fungicide. In conclusion, this study revealed moderate antioxidant property and promising antifungal activity of *A. calcarata* rhizomes.

**Table 1:** Mean scavenging concentrations of (EC50) of essential oil of *Alpinia calcarata* rhizomes in DPPH free radical scavenging assay

Sample	EC50 (μg/mL)
EO	45.8 ± 0.4a
BHT	8.4 ± 0.2b

Values are expressed as mean ± SE. The values marked with the different letters (a and b) are significantly (*P* ≤ 0.05) different from each other. BHT, butylated hydroxyl toluene; SE, standard error.

**Table 2:** Antioxidant index percentage of essential oil *Alpinia calcarata* rhizomes in TBARS assay

Sample	AI (%)
EO	16.1 ± 0.2^a^
BHT	34.7 ± 0.2^b^
Vitamin	E 32.0 ± 0.2^b^

Values are expressed as mean ± SE. The values marked with the different letters (a and b) are significantly (*P* ≤ 0.05) different from each other. BHT, butylated hydroxyl toluene; SE, standard error.

**Table 3:** Antifungal activity of essential oil of *Alpinia calcarata* rhizome

Fungi	Sample	Concentration (ppm)	Growth rate (diameter of colony in mm)				
			3 days	4 days	5 days	6 days	7 days
*Curvularia* spp.	Essential oil	1000	9.00±0.47* (73.0)	10.50±0.41* (73.0)	11.17±0.27* (76.5)	12.33±0.14* (77.4)	13.33±0.14* (79.1)
		1500	6.83±0.14* (79.5)	8.17±0.14* (80.0)	9.33±0.14* (80.3)	10.67±0.14* (80.4)	12.17±0.14* (80.9)
		2000	6.00* (82.0)	6.00* (85.2)	6.00* (87.4)	6.00* (89.0)	6.00* (90.6)
		2500	6.00* (82.0)	6.00* (85.2)	6.00* (87.4)	6.00* (89.0)	6.00* (90.6)
	Daconil	1000	15.67±0.27* (53.0)	18.83±0.36* (53.5)	23.00±0.41* (51.6)	25.67±0.27* (52.8)	28.67±0.27* (55.0)
		1500	14.83±0.14* (55.5)	17.67±0.14* (56.4)	21.17±0.14* (55.4)	24.17±0.14* (55.6)	27.33±0.27* (57.1)
		2000	14.17±0.14* (57.5)	17.17±0.14* (57.6)	20.83±0.14* (56.1)	23.50±0.24* (56.9)	26.83±0.14* (58.9)
		2500	13.83±0.14* (58.5)	16.67±0.14* (58.8)	19.83±0.14* (58.2)	23.17±0.14* (57.5)	26.17±0.14* (58.9)
	Control		33.33±0.27	40.50±0.24	47.50±0.41	54.50±0.24	63.67±0.72
	Blank		36.67±0.27	44.83±0.36	51.67±0.27	58.50±0.24	67.67±0.27
*Colletotrichum* spp.	Essential oil	1000	20.50±0.24* (42.0)	24.83±0.14* (44.0)	29.33±0.14* (46.0)	33.83±0.14* (46.9)	38.67±0.27* (46.8)
		1500	17.67±0.14* (50.0)	21.33±0.27* (51.9)	26.17±0.14* (51.8)	30.17±0.14* (52.6)	34.17±0.14* (53.0)
		2000	12.33±0.27* (65.1)	15.50±0.24* (65.0)	20.17±0.14* (62.9)	23.67±0.14* (62.8)	27.33±0.27* (62.4)
		2500	9.50±0.24* (73.1)	11.67±0.14* (73.7)	14.17±0.14* (73.9)	16.83±0.14* (73.6)	19.67±0.14* (72.9)
	Daconil	1000	17.67±0.27* (50.0)	20.83±0.36* (50.0)	24.83±0.36* (54.3)	29.00±0.41* (54.4)	33.67±0.27* (53.7)
		1500	16.17±0.14* (54.2)	19.17±0.14* (54.2)	22.17±0.14* (59.2)	25.17±0.14* (60.5)	29.67±0.27* (59.2)
		2000	14.83±0.14* (58.0)	17.83±0.14* (58.0)	20.50±0.24* (62.3)	22.83±0.14* (64.1)	26.50±0.24* (63.5)
		2500	12.67±0.14* (64.1)	16.33±0.27* (64.1)	18.50±0.24* (65.9)	20.83±0.14* (67.3)	23.83±0.14* (67.2)
	Control		35.33±0.27	44.33±0.27	54.33±0.27	63.67±0.27	72.67±0.27
	Blank		38.67±0.27	49.67±0.27	59.00±0.47	69.67±0.27	78.67±0.72

* Significant at *P* ≤ 0.05 level with the control or blank; diameter of circular fungi culture disc = 6.0 mm.
